# Erythrocyte aldose reductase activity and sorbitol levels in diabetic retinopathy

**Published:** 2008-03-24

**Authors:** G. Bhanuprakash Reddy, A. Satyanarayana, N. Balakrishna, Radha Ayyagari, M. Padma, K. Viswanath, J. Mark Petrash

**Affiliations:** 1 National Institute of Nutrition, Indian Council of Medical Research, Hyderabad, India; 2 Department of Ophthalmology and Visual Sciences, University of Michigan, Ann Arbor, Michigan; 3 Sarojinin Devi Eye Hospital and Institute of Ophthalmology, Hyderabad, India; 4 Department of Ophthalmology and Visual Sciences, Washington University, St. Louis, Missouri

## Abstract

**Purpose:**

Activation of polyol pathway due to increased aldose reductase (ALR2) activity has been implicated in the development of diabetic complications including diabetic retinopathy (DR), a leading cause of blindness. However, the relationship between hyperglycemia-induced activation of polyol pathway in retina and DR is still uncertain. We investigated the relationship between ALR2 levels and human DR by measuring ALR2 activity and its product, sorbitol, in erythrocytes.

**Methods:**

We enrolled 362 type 2 diabetic subjects (T2D) with and without DR and 66 normal subjects in this clinical case-control study. Clinical evaluation of DR in T2D patients was done by fundus examination. ALR2 activity and sorbitol levels along with glucose and glycosylated hemoglobin (HbA1C) levels in erythrocytes were determined.

**Results:**

T2D patients with DR showed significantly higher specific activity of ALR2 as compared to T2D patients without DR. Elevated levels of sorbitol in T2D patients with DR, as compared to T2D patients without DR, corroborated the increased ALR2 activity in erythrocytes of DR patients. However, the increased ALR2 activity was not significantly associated with diabetes duration, age, and HbA1C in both the DR group and total T2D subjects.

**Conclusions:**

Levels of ALR2 activity as well as sorbitol in erythrocytes may have value as a quantitative trait to be included among other markers to establish a risk profile for development of DR.

## Introduction

About 200 million people across the globe are estimated to have diabetes of which Southeast Asia alone is home to 46.9 million diabetics and India has 41 million diabetics [1,2]. Type 2 diabetes (T2D) accounts for roughly 90 percent of all diagnosed cases of diabetes [2]. The prevalence of diabetes in India is estimated to be between 5.9%–24.2% (average of 12.1%) [3,4]. It is higher in developed countries compared to developing countries. While in developing countries the average age of people with diabetes is between 45 and 64 years of age, in developed nations it is 65 years and older. These statistics indicate that the world, particularly India, is facing a growing diabetes epidemic of potentially devastating proportions. Prolonged exposure to chronic hyperglycemia, without proper management, can lead to various short-term and long-term secondary complications, both of macro and microvascular nature, which represent the main cause of morbidity and mortality in diabetic patients [5]. Hyperglycemia is the major determinant of microvascular complications in diabetes [6,7].

Diabetic retinopathy (DR), a vascular disorder affecting the microvasculature of the retina, is a leading cause of adult blindness and is the most common complication of diabetes [8]. It is estimated that DR develops in more than 75% of diabetics who have had diabetes for 15–20 years. It is projected that by 2005, diabetes will affect 300 million people worldwide, of whom 10% will develop visual impairment secondary to DR [9]. While a study reported the prevalence of DR among diabetic subjects (both rural and urban) in India was 10% [10], another study reported the prevalence of DR about 17% among urban diabetic subjects in India [11]. In a clinical study the prevalence of DR was 34% among T2D patients [12]. The prevalence of DR was 0.5% in the general rural populations of Southern India (this in total population but not among diabetics) and 10.5% among diabetic patients [13].

Although the exact mechanism involved in the pathogenesis is not known, many biochemical pathways associated with hyperglycemia have been implicated in the development of diabetic complications including DR. These include glucose autoxidation, polyol pathway, prostanoid synthesis, protein glycation, protein kinase C activation, and the hexosamine pathway [5]. Among these, the polyol pathway has been extensively studied. Aldose reductase (ALR2; EC: 1.1.1.21), the first and rate-limiting enzyme in the polyol pathway, reduces glucose to sorbitol using nicotinamide adenine dinucleotide phosphate (NADPH) as a cofactor. Sorbitol is then metabolized to fructose by sorbitol dehydrogenase [14]. Studies on animal models of diabetes and galactosemia suggest increased polyol pathway activity in the pathogenesis of DR [15]. Further, several studies based on specific inhibitors of ALR2 support the role of polyol pathway in the pathology of DR [16-18]. Retinal capillary pericytes contain ALR2, and the accumulation of polyols in pericytes has been linked to their degeneration and selective death [19]. Pericyte loss, the major event of early DR, has been observed in galactose-fed dogs that developed retinopathy [20]. In addition, the involvement of ALR2 in DR has been recently supported by the findings that ALR2 inhibitor prevented a spectrum of neural, glial, and vascular abnormalities associated with development of DR in rat and humans [17,18]. Furthermore, evidence for the involvement of ALR2 as a risk factor for DR and other diabetic complications comes from genetic polymorphism studies [21,22]. Several studies indicate that the Z-2 allele and a putative protective allele, Z+2, of ALR2 are significantly associated with DR [21-24]. Although, most animal studies with ALR2 inhibitors (ARI) have yielded encouraging results (considering some inconsistent data), on the whole, clinical trials of ARI have failed to shown efficacy against various diabetic complications. This may be due, in part, to differences in tissue levels of ALR2 in rodents as compared with humans. In addition, development of diabetic complications in humans may be influenced by metabolic and signaling pathways that have a less significant impact on the pathogenesis of complications in animal models. In principle, all diabetic patients might be expected to develop diabetic microvascular complications if hyperglycemia alone were the triggering factor for activation of the polyol pathway. Multiple factors are likely to be involved in predisposing diabetic subjects to DR, as evidenced by the fact that many, but not all, diabetic patients develop one or more microvascular complications. It has been reported that the prevalence of DR is associated with increased erythrocyte ALR2 protein levels [25]. This observation is based on previous studies that revealed that erythrocyte ALR2 protein levels correlates with ALR2 protein levels in retinal cells, particularly pericytes [25,26]. However, it was shown that ALR2 in human erythrocytes exists in activated and unactivated forms, and in hyperglycemia the total activity of ALR2 increases [15,27]. Hence, correlating total ALR2 activity with DR prevalence may provide an important link between an easily measurable marker in peripheral blood and risk of progression toward eye disease. In this study, we examined the activity of ALR2 in erythrocytes obtained from diabetic patients with and without retinopathy. Further, we also measured the levels of sorbitol as a surrogate marker for ALR2 activity levels in erythrocytes. These data demonstrate that erythrocyte ALR2 activity and sorbitol levels are significantly elevated in diabetic patients with retinopathy as compared with diabetics without retinopathy or patients without diabetes.

**Table 1:** Clinical and biochemical features of the study subjects

**Group/** **parameter**	**Nondiabetic**		**Diabetes without retinopathy**		**Diabetic retinopathy**	
Male	Female	Male	Female	Male	Female
Age (Years) Mean *n* S.D.	54.37 43 12.925	56.00 23 12.803	50.91 64 11.394	49.09 100 9.242	53.09 122 10.188	54.49 76 7.431
Glucose (mg/dL) Mean *n* S.D.	110.50 43 25.060	104.22 23 17.9	210.92 64 111.06	217.91 100 101.50	244.15 115 97.70	251.22 76 106.73
Duration (Years) Mean *n* S.D.	0.00 43 0.000	0.00 23 0.000	5.26 64 4.200	6.79 100 4.850	9.43 115 6.073	11.04 76 6.673
HbA1C (%) Mean *n* S.D.	5.20 20 1.77	5.11 13 1.27	7.90 14 1.95	7.48 26 2.26	8.40 42 2.64	8.24 24 1.68
ALR2 (units/g Hb) Mean *n* S.D.	2.49 43 1.60	3.6 23 2.3	3.60 64 2.23	3.5 100 2.36	4.62 122 3.05	4.67 76 2.69
Sorbitol (μg/mL) Mean *n* S.D.	2.9 14 0.99	3.0 10 1.4	3.4 10 1.4	3.8 27 1.0	4.5 26 1.7	5.3 17 2.3

The distribution of age, random glucose, duration of diabetes, glycosylated hemoglobin (HbA1C), ALR2 activity and sorbitol levels between male and females in nondiabetic control, diabetes without retinopathy (DNR) and diabetes with retinopathy (DR) groups. The data (mean ± standard deviation (SD)) indicate no significant difference between the genders. (n = number).

## Methods

### Subjects and study design

A hospital-based prospective case control study was conducted. The study protocols were approved by the Institutional Ethics Committees of the institutes involved. Subjects were recruited from the patients who visited the Sarojini Devi Eye Hospitals and Institute of Ophthalmology, Hyderabad, India and Department of Endocrinology, Osmania General Hospital, Hyderabad, India. A total of 362 T2D subjects (198 with DR, 164 without ocular complications) and 66 normal subjects were investigated. Written consent was obtained from the participants after they were given an explanation of the study details. A complete history of each participant, with respect to age, gender, clinical symptoms, diabetes type and duration, medication, and socioeconomic background, was collected using a well designed questionnaire. None of the diabetics in this study were on insulin treatment. The fundus of each subject was evaluated by both direct and indirect ophthalmoscopy, and DR was defined and classified according to Viswanath and McGavin [28]. The presence of retinal microaneurysm, dot and slot hemorrhages, intraretinal microvascular abnormalities, and cotton wool spots were defined as nonproliferative DR (NPDR), which was then categorized as mild, moderate, severe, and diabetic maculopathy. Formation of new vessels with and without bleeding and production of vitreous hemorrhage was defined as proliferative DR (PDR).

**Figure 1:**
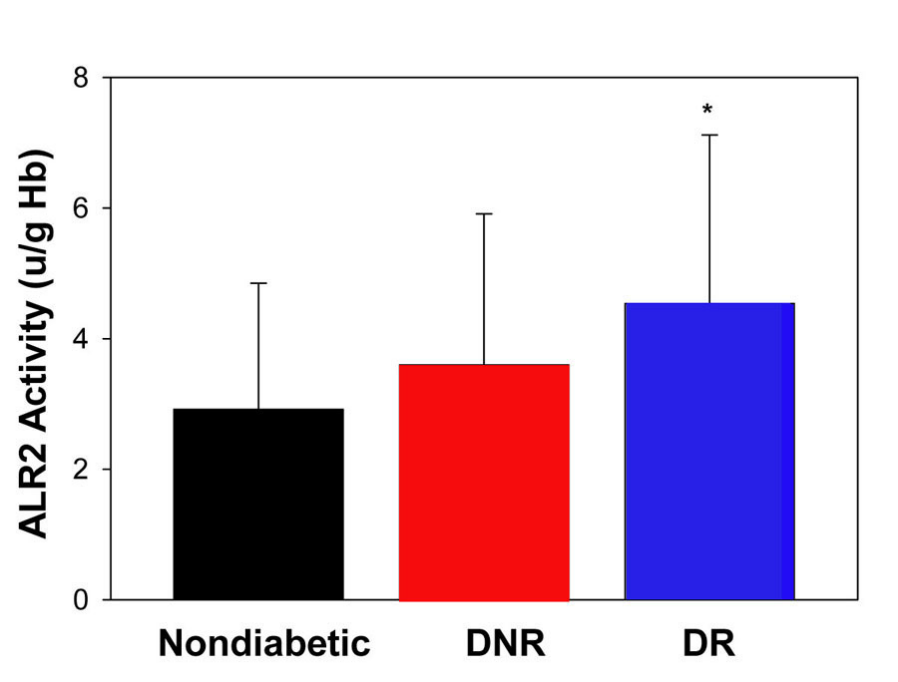
Erythrocyte aldose reductase activity. Data represent mean ± standard deviation of aldose reducatase (ALR2) activity in nondiabetic control (n=66) and diabetics without diabetic retinopathy (DNR; n=164) and those with diabetic retinopathy (DR; n=182). Asterisk (*) designates statistical significance (p<0.05) in comparison to the other groups.

### Sample collection and processing

Blood was drawn from the subjects into anticoagulant tubes and immediately transported to the laboratory on ice. Red blood cells (RBC) were separated by centrifugation, washed thrice with saline, and stored at –85 °C until further analysis.

### Glucose estimation

Glucose was estimated in plasma by the GOD-POD method using a kit (BioSystems, Barcelona, Spain). Ten µl of serum or standard (100 mg/dl glucose) was added to reagent A (100 mM phosphate buffer, pH 7.5 with 5 mM phenol, 10 U/ml glucose oxidase, 1 U/ml peroxidase, and 0.4 mM 4-aminoantipyrine) and incubated for 5 min at 37 ^o^C. Absorbance was measured at 505 nm in a spectrophotometer.

**Figure 2:**
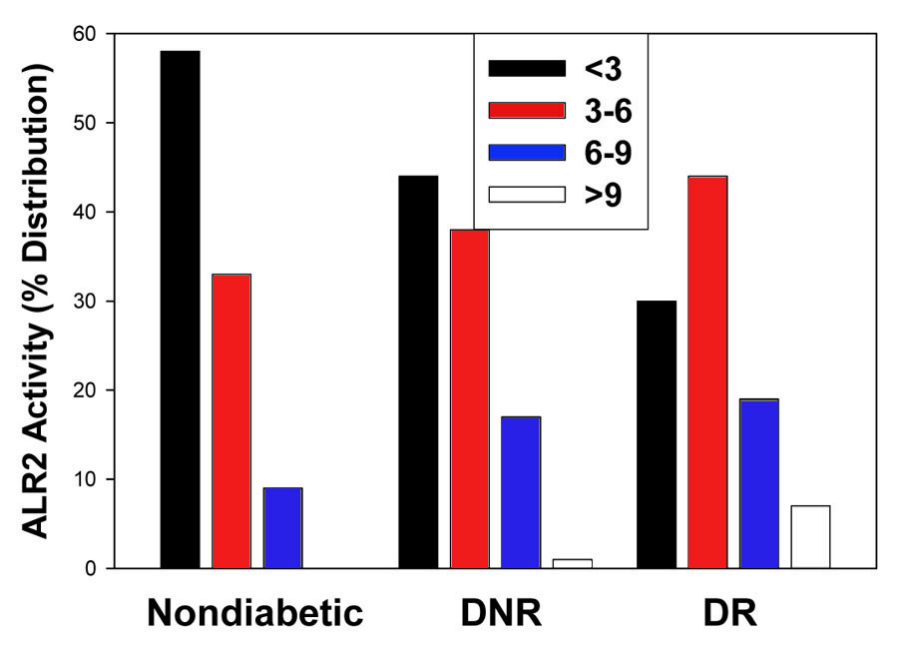
Percentage distribution of aldose reductase activity levels. Aldose reductase (ALR2) activity is distributed into <3, 3–6, 6–9, and >9 units/g Hb subgroups in nondiabetic control, and diabetics without diabetic retinopathy (DNR), and diabetics with diabetic retinopathy (DR). Percentage distribution of ARL2 activity with <3 units is significantly (p<0.05) different between the groups. Percentage distribution of ARL2 activity with >9 units is significantly (p<0.05) different between DNR and DR groups.

### Glycosylated hemoglobin

HbA1C was estimated by ion-exchange chromatography using a kit (BioSystems). Fifty µl of blood was added to reagent 1 (50 mM potassium phosphate, pH 5.0 with 5 g/l detergent and 0.95g/l sodium azide) and mixed thoroughly to prepare the hemolysate. From this 50 µl of hemolysate was added to the ion-exchange resin and washed with 2.2 ml of reagent 2 (30 mM potassium phosphate, pH 6.5 containing 0.95 g/l sodium azide). HbA_1_C was eluted using reagent 3 (72 mM potassium phosphate, pH 6.5 containing 0.95 g/l sodium azide). Absorbance of the eluted HbA_1_C was read at 415 nm in a spectrophotometer.

### Aldose reductase activity

A 10% erythrocyte suspension was made by adding 50 mM sodium phosphate buffer, pH 7.4, containing 150 mM NaCl. The suspension was lysed by repeated freezing and thawing (three cycles) and centrifuged to remove insoluble material, if any. ALR2 activity was measured spectrophotometrically using an appropriately diluted hemolysate according to a previously described method [29] using a SpectraMax spectrophotometer (Molecular Devices, Sunnyvale, CA). One unit was defined as micromoles NADPH oxidized/g Hb/ min. The assay mixture in 1 ml contained 50 µmol potassium phosphate buffer pH 6.2, 0.4 mmol lithium sulfate, 5 µmol 2-mercapto ethanol, 10 µmol DL-glyceraldehyde, 0.1 µmol NADPH and enzyme preparation (hemolysate). The assay mixture was incubated at 37 ^o^C and initiated by the addition of NADPH at 37 ^o^C. The change in the absorbance at 340 nm due to NADPH oxidation was followed.

### Estimation of sorbitol

Sorbitol was extracted by homogenizing RBC in nine volumes of 0.8 M perchloric acid. The homogenate was centrifuged at 5,000g at 4 °C for 10 min, and the pH of the supernatant was adjusted to 3.5 with 0.5 M potassium carbonate. The sorbitol content of the supernatant was measured by fluorometric method as described previously [30] using a fluorometer (Jasco-FP-6500, Tokyo, Japan). One ml reaction mixture, consisted of 50 µmol glycine buffer, pH 9.4, 2 µmol magnesium chloride, 0.2 µmol nicotinamide adenine dinucleotide (NAD) and protein-free supernatant, was incubated for 5 min at 37 ^o^C and reaction was initiated by the addition of 0.6 U of sorbitol dehydrogenase. The relative fluorescence due to NADH formation was measured in a fluorometer with an excitation wavelength at 360 nm and an emission wavelength of 452 nm. Sorbitol standards, ranging from 0.2-9.0 µg/ml, were analyzed by the same to generate a standard curve.

### Statistical analysis

The data were expressed as mean ± standard deviation. Mean values were compared by one-way ANOVA with post hoc tests of least significant difference method. Differences between comparison groups were considered to be significant where p<0.05. Correlations were calculated to study relationship of ALR2 and sorbitol with other variables. *P* values were also calculated for ALR2 in these groups.

**Figure 3:**
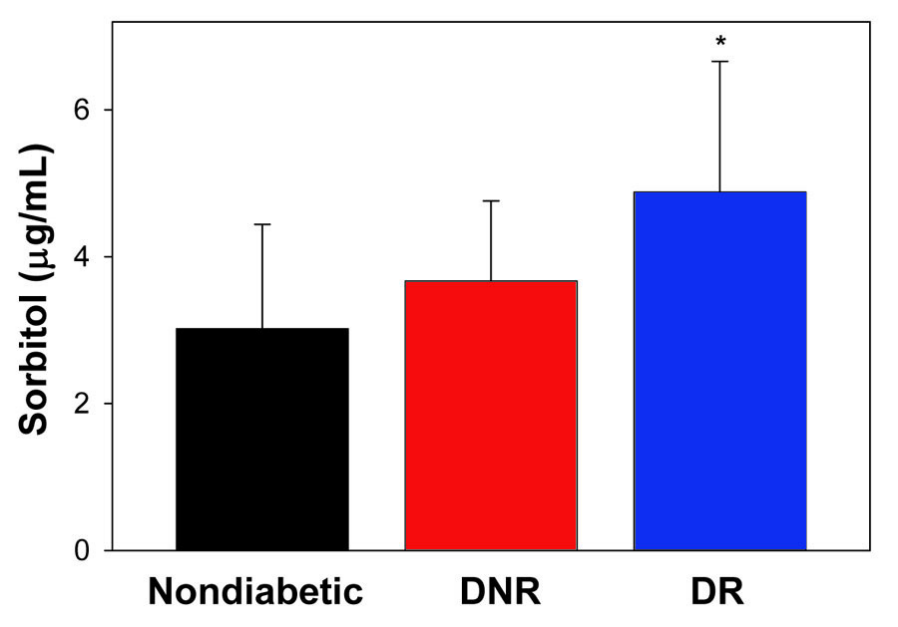
Erythrocyte sorbitol levels. Data represent mean ± standard deviation in nondiabetic control (n=31) and diabetics without diabetic retinopathy (DNR; n=44) and those with diabetic retinopathy (DR; n=52). Asterisk (*) designates statistical significance (p<0.05) in comparison to the other groups.

## Results

Data on mean age, duration of diabetes, levels of glucose, glycosylated hemoglobin, ALR2 activity, and sorbitol with respect to gender distribution for nondiabetic control, diabetics without retinopathy (DNR) and diabetics with retinopathy (DR) groups are summarized in Table 1. There was no significant difference (p>0.05) between male and female subjects in all three groups with respect to the measured parameters. Therefore, the pooled data for men and women in respective groups were considered for subsequent analysis.

As can be seen from Figure 1, erythrocyte ALR2 activity in the DNR group was not significantly different from nondiabetic control (p>0.05). Interestingly, ALR2 activity in the DR group was significantly different not only from the control group but also from the DNR group (p<0.05) (Figure 1). However, the ALR2 activity ranged from 0.2 units/g Hb in the control group to 18.6 units/g Hb in the DR group with considerable overlap between the groups. Therefore, we examined the data after distribution of individuals into one of four subgroups defined according to level of ALR2 activity (Figure 2). Percentage distributions of ALR2 activity indicated that most subjects in the control group had <3.0 units (58%), about 33% had 3–6 units, and only 9% had activity in the category of 6–9 units. Approximately 43% of the subjects in DNR group had <3.0 units, 35% had 3–6 units, and about 18% had ALR2 activity in the category 6–9 units. Whereas most of the DR subjects (46%) had 3–6 units of ALR2 activity, a substantial proportion (20%) had 6–9 units activity, and those with >9.0 units of ALR2 activity were found predominantly in this group (6%). These results suggest that prevalence of DR is associated with higher ALR2 activity. However, there was no significant difference in ALR2 activity between NPDR and PDR (4.36 units; n=114 vs 4.78; n=84).

**Figure 4:**
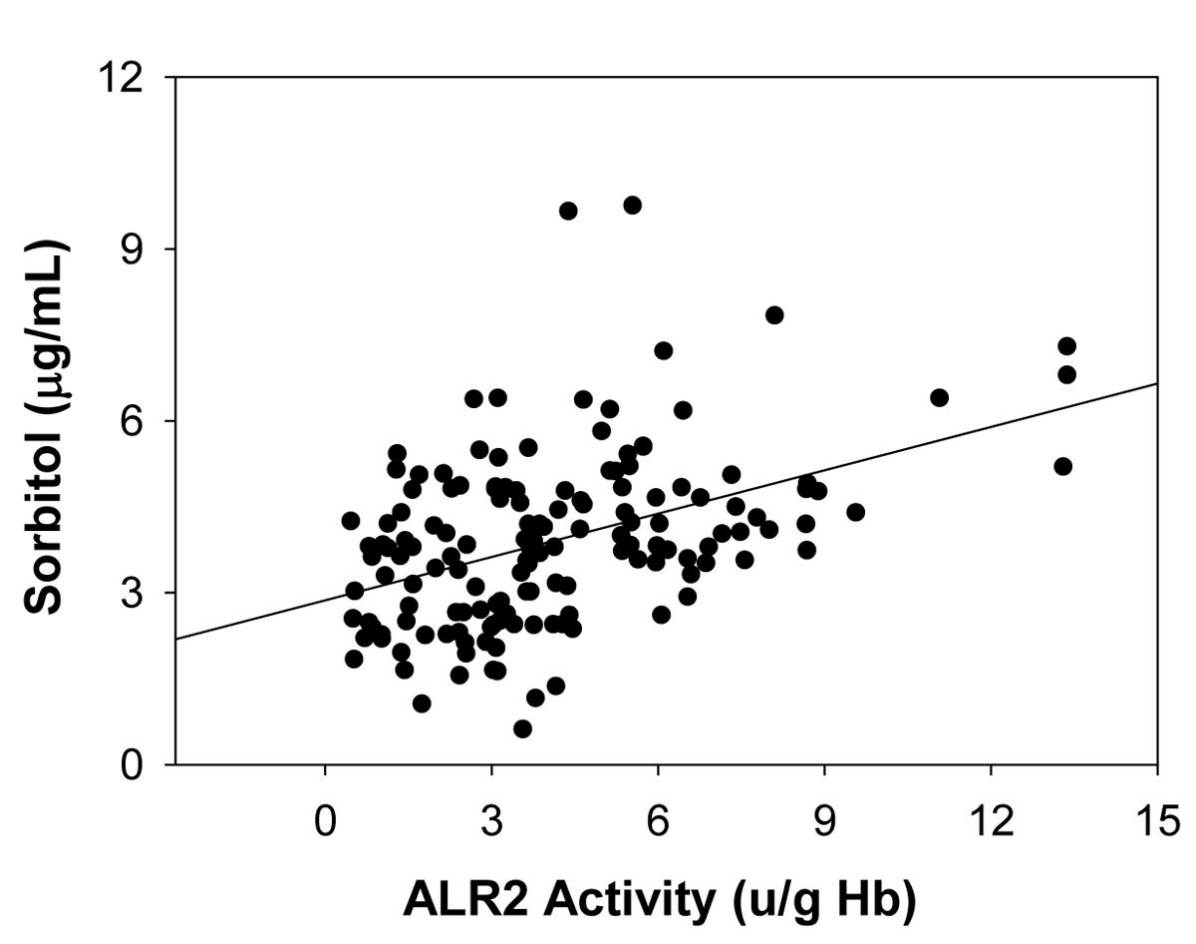
Correlation between erythrocyte sorbitol levels and aldose reductase activity. Correlation (r=0.188) between erythrocyte sorbitol levels and aldose reductase (ALR2) activity in control, diabetics without diabetic retinopathy (DNR), and diabetics with retinopathy (DR) was found to be significant at p<0.05. Correlation was done for those samples in which sorbitol was determined.

We further measured the levels of sorbitol, the product of ALR2-mediated reduction of glucose, in a subset of subjects in all three groups. While the levels of sorbitol were found to be higher in the DNR group as compared to control group, the difference was not statistically significant (p>0.05). However, sorbitol levels were significantly (p<0.05) higher in the DR group as compared to both the DNR and the control groups (Figure 3). Increased levels of sorbitol in DR patients were consistent with the higher ALR2 activity in DR patients. Activity of ALR2 was not correlated with age, glucose, diabetes duration, and HbA1C levels in all three groups (control, DNR, and DR; see Table 2) as well as with pooled data. Similarly, levels of sorbitol did not correlate with age, glucose, diabetes duration, and HbA1C levels in all three groups (Table 3) as well as with pooled data. However, as Figure 4 demonstrates, ALR2 activity was correlated with sorbitol levels (r=0.188; p<0.05) . As with ALR2 activity, there was no significant difference in sorbitol levels between NPDR and PDR subjects (4.80 mg/mL; n=41 vs 5.02; n=27).

**Table 2:** Correlation of aldose reductase activity with other clinical variables

Variables	Correlation coefficient values		
Nondiabetic	Diabetes without retinopathy	Diabetic retinopathy
Age	−0.181	−0.015	0.047
Glucose	0.030	−0.021	0.001
Duration	—	−0.049	0.016
HbA1C	0.092	−0.177	0.051

Correlation of aldose reductase (ALR2) activity with age, glucose, diabetes duration, and glycosylated hemoglobin (HbA1C) in different groups indicate all these correlates are not significant with ALR2 activity at p>0.05.

## Discussion

Although uncontrolled hyperglycemia is the major factor, the link between diabetes and susceptibility to various secondary complications has not been unraveled. The polyol pathway of glucose metabolism is activated when intracellular glucose levels are high [31]. The activation is immediately linked to hyperglycemia and occurs prominently in tissues that develop complications [31,32]. In addition, polymorphisms associated with regions flanking the ALR2 gene have been implicated in human susceptibility to DR and other diabetic complications [21,22]. There is also a strong evidence to show that diabetic complications including DR are associated with increased oxidative stress [5], and activation of polyol pathway is known to contribute to oxidative stress [33]. Evidence for the involvement of ALR2 in DR comes from studies that demonstrated ALR2 was present in different cell types of retina [17-20]. In addition, disruption of the ALR2 gene leads to a reduction in lesions associated with DR in a diabetic mouse model [34]. However, studies with ARI have yielded inconsistent results against DR or diabetic-like retinopathy in experimental animals [14,35] and in clinical trials to assess efficacy against various diabetic complications [36]. Nevertheless, ALR2 remains an intriguing candidate/target for the treatment of secondary complications. Hence, the role of the polyol pathway vis á vis ALR2 in the pathogenesis of diabetic complications, particularly DR, requires further investigations from various angles.

The present study investigated the functional state of erythrocyte ALR2 in DR patients in comparison with DNR. The results demonstrated that the activity of ALR2 is significantly higher in DR patients as compared to DNR patients. Earlier studies had focused on enzyme activity and protein levels of ALR2 in diabetic complications in humans [reviewed in 22]. In type 1 diabetes, patients with the highest ALR2 activity were found to be four times more likely to develop diabetic microvascular complications than those whose activity was similar to normal. Oishi et al. suggested that increased prevalence of DR is correlated with increased erythrocyte AR protein levels, particularly the prevalence of DR in patients who have diabetes for fewer than 10 years [25]. A correlation between erythrocyte ALR2 protein levels and diabetic cataract, particularly posterior subcapsular cataract, has also been reported [37]. However, in these studies ALR2 levels were determined by ELISA and not by catalytic activity. The underlying assumption behind these studies was that erythrocyte ALR2 might reflect ALR2 in pericytes and lens, the vulnerable cell types in DR and cataract, respectively. The best way to assess the functional role of ALR2 in DR is to determine ALR2 in retinal pericytes and capillaries. However, a noninvasive procedure is not currently available to make such measurements. Therefore, we and others used erythrocytes as a surrogate tissue for enzyme measurements.

Increased oxidative stress has been linked to the development of diabetic complications [5] and altered redox homeostasis is known to affect ALR2 activity. For example, oxidation of cysteine residue under oxidative conditions modulates ALR2 activity [38]. Thus the specific activity of the protein may also be critical for the development of diabetic complications. The results of the present study show that higher activity of ALR2 is associated with prevalence of DR. On the other hand, we found no significant difference in ALR2 activity between NPDR and PDR, indicating higher ALR2 might be involved in the initiation of disease, but not in the progression, which needs further investigation. However, there was a large variation in ALR2 activity, and there was considerable overlap of activity between nondiabetic control, DNR, and DR groups. Nevertheless, percentage distributions of ALR2 activity indicate that a substantial number of subjects in the DR group had activity in the category of 6–9 units, and, most important, >9.0 units of ALR2 activity was found almost exclusively in this group.

**Table 3:** Correlation of sorbitol levels with other clinical variables

Variables	Correlation coefficient values		
Nondiabetic	Diabetes without retinopathy	Diabetic retinopathy
Age	0.067	−0.033	−0.146
Glucose	0.087	0.012	−0.047
Duration	—	−0.208	−0.015
HbA1C	0.047	0.297	0.130

Correlation of sorbitol levels with age, glucose, diabetes duration, and glycosylated hemoglobin (HbA1C) in different groups indicate that all these correlates are not significant with sorbitol levels at p>0.05.

Development of DR and other microvascular complications are generally linked to diabetes duration and patient age [25,39-41]. It is estimated that DR develops in more than 75% of diabetics who have had diabetes for 15–20 years. Interestingly, the higher ALR2 activity in the DR group in the present study was not associated with diabetes duration and patient age. This observation suggests that ALR2 activity might serve as an independent risk identification factor for DR, irrespective of duration of diabetes and age of the patient. In this study, we found a substantial number of DR patients whose duration of diabetes was less than 15 years: Of 198 DR patients in our study, 28% had been diabetic for <5 years, 35% for <10 years, and 35% for >15 years. This may have implications in the development of DR in Indian context. Even though the prevalence of microvascular complications of diabetes-like retinopathy is comparatively lower in the Indian populations, the age of onset of diabetes in this part of the world is much earlier [2,42]. The impact of early onset diabetes and development of complications with shorter duration of diabetes needs to be addressed. We also note that, in contrast to studies [41,43,44], we did not observe an association between HbA1C and prevalence of DR in our study population. Furthermore, the increase in ALR2 activity in DR group was not associated with HbA1C levels. A previous study also reported that while the protein level of ALR2 in erythrocyte was associated with DR, there was no correlation between enzyme levels and age, duration of diabetes, fasting blood glucose, and HbA1C in patients with T2D [45]. The interindividual variability of ALR2 protein content has been shown to be associated with variation in polyol pathway metabolites [46], which could be associated, at least in part, to the prevalence of diabetic complications. A significant proportion (30%) of DR patients in our study were shown to have >6 units of ALR2 activity, whereas most of the DNR patients and nondiabetics had <6.0 units activity. Although relatively few of our study participants had ALR2 activity above 9.0 units, the strong association of these high enzyme levels with the DR group suggests that elevated erythrocyte ALR2 activity may represent a significant risk factor for the susceptibility of diabetic subjects to develop DR. Further, the possibility that patients in DNR group with 6–9 units activity might eventually develop DR needs further investigation.

Measurement of ALR2 activity by the spectrophotometric method using DL-glyceraldehyde as the aldehyde substrate could lead to confusing results because other aldo-keto reductases are active with this substrate [47]. Thus, we measured sorbitol level in a subset of samples as an index of ALR2 activity since ALR2 is unique among human aldo-kedo reductases in its ability to catalyze the NADPH-dependent conversion of glucose to sorbitol [48]. As with ALR2 activity, the sorbitol levels were significantly higher in the DR group as compared to the control and DNR groups (Figure 3). It should be noted that there was linear correlation between sorbitol levels and ALR2 activity irrespective of the group to which they belonged (Figure 4). Moreover, the contribution of other aldo-keto reductases to the apparent ALR2 activity is likely to be minimal because of the good correlation between apparent ALR2 activity levels and sorbitol levels in RBC. In addition, sorbitol levels were not associated with diabetes duration, age, glucose, and HbA1c levels of the subjects. As with ALR2 activity, we observed that sorbitol levels were also not related to the severity of DR, as there was no significant difference between NPDR and PDR subjects.

Based on the results of the current study, it is reasonable to hypothesize that some diabetics with ALR2 activity levels above a certain “threshold” level might be predisposed to develop DR. However, validation of this hypothesis will require additional longitudinal studies. Additional studies are needed to determine whether ALR2 activity and sorbitol levels in erythrocytes may have value as quantitative traits that need to be considered, along with other known risk factors (including genetic susceptibility), to assess risk for development of DR.

Although the beneficial impact of strict glycemic control on prevention of diabetic complications has been well established, most individuals with diabetes rarely achieve consistent euglycemia. Hence, agents that can substantially delay or prevent the onset and development of diabetic complications, irrespective of glycemic control, would offer many advantages. In principle, ARI can be included in this category. Although clinical trials of ARI have failed to demonstrate efficacy against various diabetic complications, trials of other compounds such a protein kinase Cβ inhibitor have also failed to show efficacy against progression of DR [49]. Thus, intensive research continues to identify and test both synthetic as well as natural products for their therapeutic value to prevent the onset as well as progression of diabetic complications [29,50-52].
